# Optimization and audit of radiation dose during percutaneous transluminal coronary angioplasty

**DOI:** 10.4103/0971-6203.37478

**Published:** 2007

**Authors:** Roshan S. Livingstone, B. S. Timothy Peace, Sunil Chandy, Paul V. George, Purendra Pati

**Affiliations:** Department of Radiology, Christian Medical College, Vellore, Tamil Nadu, India; * Department of Cardiology, Christian Medical College, Vellore, Tamil Nadu, India

**Keywords:** Dose area product, radiation dose

## Abstract

The percutaneous transluminal coronary angioplasty (PTCA) is one of the interventional procedures which impart high radiation doses to patients compared to the other cardiologic procedures. This study intends to audit and optimize radiation dose imparted to patients undergoing PTCA. Forty-four patients who underwent PTCA involving single or multiple stent placement guided under cardiovascular X-ray machine were included in the study. Radiation doses were measured using dose area product (DAP) meter for patients undergoing single and multiple stent placements during PTCA. A dose reduction of 27-47% was achieved using copper filters and optimal exposure parameters. The mean DAP values before optimization were 66.16 and 122.68 Gy cm^2^ for single and multiple stent placement respectively. These values were 48.67 and 65.44 Gy cm^2^ respectively after optimization. In the present scenario, due to the increase in the number of PTCAs performed and the associated risk from radiation, periodical audit of radiation doses for interventional procedures are recommended.

Cardiovascular interventions are complex procedures performed with dedicated fluoroscopy machines. In recent times, these interventions are rapidly replacing sizable fraction of cardiovascular surgeries. The PTCA involves applications such as stent deployment, resulting in high radiation doses due to increasingly long exposure times[1,2] The duration of PTCA depends on the complexity of the disease. The more complex the anatomy, the longer the duration of fluoroscopic screening and the number of cine runs acquired. During PTCA, the radiation doses to patients are relatively high[3–6] It is therefore prudent to perform dose auditing on this procedure by implementing radiation safety protocols, ensuring good work practices and comparing risks involved with benefits obtained. Establishing reference levels for interventional examinations presents a problem since patient numbers are limited and these interventional procedures are often performed at a few specialist centers.[7]

Radiation doses imparted to patients can be measured using a dose area product (DAP) meter. The DAP is the most reliable measurement technique for dynamic examinations such as fluoroscopy in which the projection technique and the technique parameters are continually varying.[7,8] Moreover, DAP allows the estimation of patient dose in complex examinations and does not depend on the distance from the X-ray tube.[9] The DAP is particularly useful for assessing and comparing radiation doses from screening procedures, and it provides useful indication of the overall patient exposure rather than measurement of surface dose and interprets doses to particular organs.[10] Though DAP is an ancient measurement tool, it still remains as one of the best ways to measure radiation doses from diagnostic and interventional cardiologic and radiological procedures.

Dose auditing during radiological procedures is of great value in optimizing radiation dose to patients without compromising diagnostic yield. Modification of key imaging parameters such as tube voltage, mAs, field collimation, geometric magnification and radiation exposure to the image receptor has an impact on image quality and the radiation dose to the patient and these require optimization. The present study is intended to audit and optimize radiation dose imparted to patients during PTCA procedure.

## Materials and Methods

All PTCA procedures were performed using Philips Integris H5000 (the Netherlands) machine dedicated for cardiovascular procedures. The machine incorporated preprogrammed radiographic factors to suit the region of anatomical interest. It also had several selection modes with specific table-top dose rates such as low, normal and high for fluoroscopy screening. In the initial stages of dose auditing, the table-top dose rate for ‘low fluoroscopy mode’ was set to 43.8 mGy/min, ‘normal’ to 87.5 mGy/min and ‘high’ to 175 mGy/min. The Philips H5000 machine incorporated a minimum filtration of 1.5 mm Al equivalent; and added filtration (spectral filters) of 0.1, 0.2 and 0.4 mm copper. The spectral filters were automatically selected by the machine according to the dose rate settings selected by the operator. The machine had a Charged Couple Device (CCD) camera fixed along with the image intensifier. The advantage of using CCD camera was that it necessitated lower exposure factors, thus imparting less radiation dose to patients.

The optimization process (P2*) involved halving the dose rates to 21.9, 43.8 and 87.5 mGy/min for fluoroscopy selection modes of low, normal and high respectively. This optimization was achieved by increasing the existing filtration specified by the manufacturer and by increasing tube potentials and lowering the tube current. The dose optimization techniques using copper filters are still practiced, and more literature is required in the current scenario due to the arrival of flat panel machines. Ideally, the kVp and the filter should be preferably chosen so that for the resultant X-ray spectrum, the sensitivity of the detector or detector combination should be maximum. In addition, the energy should be optimized for better visibility of narrow blood vessels with contrast agents. For the kVp and filter used in our study, cardiologists found that the images were of optimum quality for performing the procedures.

### PTCA procedure

The PTCA involves opening stenosed segments in coronary vessels using balloon angioplasty and deploying stents for long-term patency in majority of cases. After gaining femoral arterial access using the Seldinger technique, patients are taken for PTCA directly if a coronary angiogram (CA) was already available. Using dedicated guiding catheters, the coronary ostea is cannulated. After proper angiographic delineation, the optimum views are selected to show the site and length of lesion without fore shortening. The lesion is crossed with 0.014 inch sized wires and dilated with appropriate sized balloons. Adequately sized premounted stents are then deployed at the lesion site for long-term patency. Final cine runs in orthogonal views confirmed the adequacy of the procedure. The PTCA was performed by one consultant cardiologist and one resident cardiologist. The PTCA was categorized into two groups namely patients who had a single stent placement and patients who had multiple stent placement.

It is possible to use one of the three image intensifier formats (IIFs) or field sizes with diameters such as 23, 17 and 14 cm available on both the machines. A 23-cm IIF was used during fluoroscopic screening in PTCA procedure for tracing the path of the catheter from the region of femoral puncture and in the cardiac valve region. The fluoroscopic screening along the femoral region while tracing the path of the catheter from the region of femoral puncture was done only if there was a problem in the smooth insertion of the wire. A 17-cm IIF was used for the oblique, caudal, cranial and lateral projections delineating the coronary anatomy during the procedure. The 14-cm IIF was not used unless there was a necessity for finer details while performing the procedure. Selection of these IIFs was at the discretion of the personnel performing the procedure.

### Dosimetry

**Figure 1:**
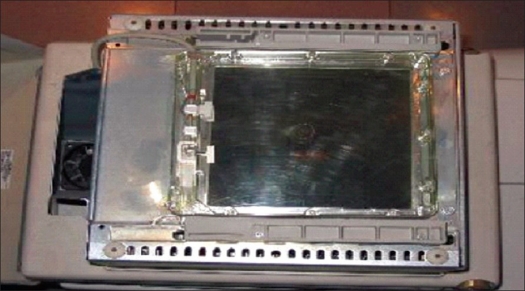
Diamentor - dose area product meter fitted on top of the collimator assembly

Radiation dose imparted to patients who underwent PTCA procedure was measured using DAP meter (diamentor PTW; Freiburg, Germany) which was fitted to the collimator assembly [Figure 1]. The DAP meter is constructed using transparent plastic, and it is therefore completely unobstructive to the examination. The DAP meter measured radiation dose contributed from fluoroscopy screening and cine runs. During the course of the examination, personnel involved in the data collection continuously monitored DAP values and this facilitated acquisition of DAP values pertaining to fluoroscopy screening and cine runs separately. Periodic calibration of the DAP meter was done by company engineers every 6 months in the presence of medical physicists.

## Results

Out of the 44 patients who underwent PTCA procedure, 6 were female patients and 38 were male patients. Table 1 shows patient-related parameters along with the exposure factors used during fluoroscopic screening and cine runs. The exposure parameters used for single stent placement and multiple stent placement during PTCA with company preset values for the H5000 Philips Integris machine are shown as P2 in Table 1. The P2* represents the optimized values used in the same machine. After the optimization, it is found that tube potentials during fluoroscopic screening were higher for P2* compared those used in P2; however, the tube current in P2* was less than that used in P2. The kVp and mA were only slightly increased for cine runs, and the tube potential was increased as for fluoroscopic screening in both P2 and P2*. The fluoroscopic time duration during the single stent placement ranged from 4.9 to 29.9 min; while for multiple stent placement, it ranged from 7.9 to 25.8 min. The PTCAs were performed under automatic brightness control, in which the tube potential and tube current were adjusted.

**Table 1:** Patient-related and exposure parameters during percutaneous transluminal coronary angioplasty

*Type*	*Examination*	*No. of cases*	*Age (Yrs)*	*Mean height (cms)*	*Mean weight (kg)*	*Mean Fluoroscopy parameters*		*Mean cine parameters*				*Fluoroscopy time in min Mean (range)*
	
						*kV*	*mA*	*kV*	*mA*	*No. of runs (range)*	*Frames (range)*		
P2	Single stent	15	57	163	64	85	21	75	622	14.4 (7−23)	1283 (570−2204)	10.99 (4.9−26.5)
	Multiple stent	6	59	167	66	93	20	75	667	23 (17−28)	2107 (1310−2605)	17.62 (11.1−25.8)
P2*	Single stent	18	50	164	64	105	6	79	723	17.2 (8−31)	1566 (689−3367)	11.57 (5−29.9)
	Multiple stent	5	53	166	71	107	6	81	740	20.8 (17−27)	1761 (1218−2522)	17.72 (7.9−24.8)

P2* - optimized values in Philips Integris 5000 machine.

Table 2 shows the DAP values for PTCA. Though the time duration of fluoroscopic screening was higher than the time duration of cine runs, the percentage of radiation dose imparted to patients from cine runs was higher than that from fluoroscopic screening. The mean DAP value for single stent placement in P2 was 66.16 Gy cm^2^, whereas the mean DAP value after optimization (P2*) was 48.67 Gy cm^2^. Similarly for multiple stent placement, the mean DAP value in P2 was 122.68 Gy cm^2^ and mean DAP value for P2* was 65.44 Gy cm^2^. The Student's ‘*t*’ test showed that the DAP value from P2 for PTCA was significantly higher than that for P2* (*P* < 0.001). Table 2 also shows the dose rates during cine runs and fluoroscopic screening. After the optimization (P2*), the use of high tube filtration and low tube currents has reduced the dose rates by about 40-50% for fluoroscopic screening and cine runs respectively and hence has an effect on the overall radiation dose imparted to patients during PTCA.

**Table 2:** Dose area product values for percutaneous transluminal coronary angioplasty before and after optimization

*Type*	*Examination*	*Fluoroscopy DAP in Gy cm^2^*	*Cine DAP in Gy cm^2^*	*Total DAP in Gy cm^2^*	*Fluoroscopy dose rate (mGy cm^2^/sec)*	*Cine dose rate (mGy cm^2^/frame)*

		*Mean ± S.D (range)*	*Mean ± S.D (range)*	*Mean ± S.D (range)*		
P2	Single stent	26.13 ± 15.9 (10.7-75)	39.87 ± 20.87 (12.9-87.9)	66.16 ± 31.3 (28.2-113.6)	40.34 (14.5-56.46)	31.57 (17.11-45.92)
	Multiple stent	47.72 ± 23.32 (24.4-75.5)	74.97 ± 34.25 (36-102.6)	122.68 ± 55.75 (60.4-160.8)	44.89 (35.36-55.43)	35.71 (22.77-46.7)
P2*	Single stent	17.88 ± 9.55 (6.9-39.2)	30.79 ± 16.9 (9-66.2)	48.67 ± 24.95 (21.3-98.1)	25.38 (14.02-41.88)	19.3 (10.21-28.7)
	Multiple stent	31.42 ± 21.46 (8.7-57.8)	34.02 ± 20.43 (13.6-52.4)	65.44 ± 42.78 (22.3-109.5)	26.82 (18.19-39.16)	18.27 (10.91-23.89)

P2* - optimized values.

## Discussion

The PTCA is an interventional cardiologic procedure involving high fluoroscopic doses and replaces a sizable fraction of cardiovascular surgeries in the current scenario. Repeated PTCAs may be required for some patients in order to achieve the desired result. In spite of being a procedure of imparting high radiation doses to patients, the benefits obtained from this procedure far outweigh the risk involved from radiation. The potential deleterious consequence of this procedure involves stochastic effects as well as deterministic effects.[11]

There are no standard protocols available for PTCA; hence it is necessary to audit and optimize radiation doses during this procedure. During the optimization process in the current study, adequate copper filtration with high tube potentials and low tube currents was selected. It has been observed that with suitable thickness of copper filtration combined with optimized exposure factors, absorbed dose during fluoroscopic procedure can be reduced with little or no loss of image quality.[12] As pointed out by Fenner *et al*., there is a reduction of 40% of absorbed dose with only minimal loss of image quality with the using 0.2 mm Cu filter.[13] Use of tube potentials of 120-125 kV instead of 90-100 kV and corresponding mAs could reduce entrance surface doses by 50% and effective dose by 43%.[14]

In the current study, the radiation doses after optimization (P2*) were found to be 27% lower for single stent placement and 47% lower for multiple stent placement compared to those in P2 using company preset values. It is also noteworthy in this context that the mean DAP value of 65.44 Gy cm^2^ for multiple stenting in P2* was comparable to the mean DAP value of 66.16 Gy cm^2^ for single stenting in P2. From Table 2 it is also noted that dose rates for cine runs and fluoroscopy screening are reduced in P2*. Since the PTCA is usually preceded by a diagnostic CA, it would be recommended to include radiation dose from CA along with that involved in PTCA also, since radiation doses have a cumulative effect. The mean DAP value for patients undergoing CA procedure before optimization was 55.86 Gy cm^2^. In a separate study conducted earlier, the mean DAP value for CA procedure was 27.71 Gy cm^2^.[15] Adding the DAP value after the optimization for corresponding patients for both CA and PTCA would therefore give 76.38 and 93.15 Gy cm^2^ for single stent and multiple stent placement respectively. No ill effects of radiation, such as deterministic or stochastic, were reported for any patient during the course of the study.

The mean DAP value after the optimization in the current study for single stent placement was 48.67 Gy cm^2^, and this lies well within the values of radiation doses reported in literature. Table 3 shows a comparison of DAP values with those from studies reported in literature.[4,8,16–21] Kuon *et al*., in their study, have optimized radiation doses up to 14.4 Gy cm^2^ for PTCA by adopting techniques like training the staff in the fluoroscopy room, using low fluoroscopy modes and by limiting the number of cine runs wherever possible to one heart cycle length.[21]

**Table 3:** DAP values for PTCA in comparison with those from studies in literature

*Authors*	*Mean DAP value (Gy cm^2^)*
Vano *et al*.[4]	87.5
Betsou *et al*.[8]	38
Broad head *et al*.[16]	72.2
Efstathopoulos *et al*.[17]	75
Van der Putte *et al*.[18]	108
Tsapaki[19]	68
NRPB[20]	63
Kuon *et al*.[21]	14.4
Current study P2* (single stent)	48.67
Current study P2* (multiple stent)	65.44

P2* - optimized values.

High radiation doses are imparted to patients who require cranio-caudal angulations since they require maximum X-ray output and involve high tube loadings. Mottled appearance was visualized in both P2 and P2* during these extensive angulations. Short cine runs were required to visualize the vessels prominently if the images gave mottled appearance during fluoroscopic screening, and this requires increase of radiation doses. However, after the optimization, the images did not produce significant mottled appearance which could degrade the diagnostic information. In the current study, the length of the procedure and the duration of fluoroscopic screening and cine runs acquired depended upon the complexity of anatomy of the patient and the number of stents needed to be implanted. The increase in the number of cine runs and frames acquired during cine runs depended on the personnel performing the procedure and complexity of the disease. A few cardiologists required higher number of frames for each run in order to visualize the vessels more prominently. Radiation doses were high during the placement of stent when high degree of tube angulations, especially cranio-caudal positions, was selected for certain patients.

The cardiovascular machines are specifically designed for interventional cardiologic procedures and have the option of using different IIFs. Appropriate selection of IIFs based on exposure parameters, dose rates and image magnification required to obtain the necessary clinical information could reduce radiation dose imparted to the patient significantly. The cardiovascular machines dedicated for cardiac procedures are invariably imported from developed countries and the default preset exposure settings are based on the patient size of those specified locations. Since the weight and body surface area of Indian patients are lower in comparison with patients of other countries, it would be advisable to set up appropriate dose modes for the Indian population. To achieve low dose levels during cardiac procedures, the image intensifier entrance dose rates could be reduced according to the body mass index of the Indian population. However, radiation-intensive angulations do not enable substantially better image quality despite increasing image intensifier dose levels.[22]

The dose reduction achieved by these methods is likely to translate into low doses to the operators as well. Further dose reduction is possible by adopting pulsed fluoro mode operation. As pointed out by Kuon *et al*., use of a 25 ps^−1^ would also impart low radiation dose to patients if adequate radiation safety standards are maintained.[22] The dose can be further reduced by using 12.5 ps^−1^ in cine mode rather than using 25 ps^−1^. Maintenance and quality assurance checks at regular intervals are mandatory. Improperly calibrated machines can also inadvertently impart large doses to patients without the knowledge of the operator.[23]

The reference DAP dose levels presently available for developed countries are 45 and 75 Gy cm^2^ for CA and PTCA respectively, and our center fulfils the above criteria for these interventional procedures. In accordance with the ALARA principle, interventionists should therefore vary cine image intensifier dose rates and may to a certain extent compromise optimal image quality in accordance with diagnostic and therapeutic requirements, documented structure, tube angulation and body mass index.[22]

## Conclusion

Though the radiation dose imparted to patients does not present any alarming situation with regard to ill effects of radiation, it would be prudent to optimize radiation dose to patients undergoing PTCA and take efforts towards achieving reduction in radiation dose to the patients. Since there is a frequent change in the various imaging modalities, reference dose levels should be audited on a time-to-time basis so as to keep the doses as low as reasonably practical. Enhanced knowledge of ‘radiation dose’-reduction techniques significantly reduces patient radiation hazards in invasive cardiology.
